# The rs61742690 (S783N) single nucleotide polymorphism is a suitable target for disrupting BCL11A-mediated foetal-to-adult globin switching

**DOI:** 10.1371/journal.pone.0212492

**Published:** 2019-02-15

**Authors:** Sayed Abdulazeez, Shaheen Sultana, Noor B. Almandil, Dana Almohazey, B. Jesvin Bency, J. Francis Borgio

**Affiliations:** 1 Department of Genetic Research, Institute for Research and Medical Consultation (IRMC), Imam Abdulrahman Bin Faisal University, Dammam, Saudi Arabia; 2 Interserve Learning and Employment, As Sawari, Al Khobar, Saudi Arabia; 3 Department of Clinical Pharmacy Research, Institute for Research and Medical Consultation (IRMC), Imam Abdulrahman Bin Faisal University, Dammam, Saudi Arabia; 4 Department of Stem Cell Research, Institute for Research and Medical Consultation (IRMC), Imam Abdulrahman Bin Faisal University, Dammam, Saudi Arabia; 5 Department of Biotechnology, Malankara Catholic College, Mariagiri, Kanyakumari District, Kaliakkavilai, Tamil Nadu, India; University of Naples Federico II, ITALY

## Abstract

**Background:**

B-cell lymphoma/leukaemia 11A (BCL11A) is a C2H2-type zinc-finger transcription factor protein that is a critical modulator of haemoglobin switching and suppresses the production of foetal haemoglobin. Variation in the *BCL11A* gene ameliorates the severity of sickle cell disease (SCD) and β-thalassemia (β-thal). The *BCL11A* gene is located on chromosome 2p16.1 and encodes an 835-amino acid protein.

**Method:**

Using state-of-the-art *in silico* tools, this study examined the most pathogenic non-synonymous single nucleotide polymorphisms (nsSNPs) that disrupt the BCL11A protein and mediate foetal-to-adult globin switching. A total of 11,463 SNPs were retrieved from the Single Nucleotide Polymorphism database (dbSNP). These included 799 in the 5′ untranslated region (UTR), 486 in the 3′ UTR, and 266 non-synonymous, 189 coding synonymous, six nonsense, and six stop-gained SNPs.

**Results and discussion:**

*In silico* tools (SIFT, SNAP, PolyPhen-2, PANTHER, I-Mutant, PROVEAN, SNPs&GO, mCSM, and PhD-SNP) predicted the five most-deleterious nsSNPs: rs61742690, rs62142605, rs17028351, rs115666026, and rs74987258. Molecular dynamic simulation and homology modelling of the mutated proteins (S783N, D643N, G451S, K670R, and M313L) of the most deleterious nsSNPs revealed their functional and structural impact. nsSNP rs61742690 was predicted to be the most deleterious, as supported by eight of the nine *in silico* tools.

**Conclusions:**

Complete failure in the protein–protein interactions with functional partners (KLF1 and others) and significant changes (±100% variation) in the interface energy revealed that rs61742690 (S783N) in the zinc-finger domain is a suitable target for disrupting BCL11A-mediated foetal-to-adult globin switching.

## Introduction

B-cell lymphoma/leukaemia 11A (BCL11A) is a transcriptional repressor of foetal haemoglobin (HbF) and an ameliorating factor in sickle cell disease (SCD) and β-thalassemia (*β-*thal). These diseases are a high burden in most developing countries [1,2]. BCL11A, which is essential for postnatal development and normal lymphopoiesis, is expressed in several hematopoietic tissues, including the bone marrow, splenic B and T cells, monocytes, megakaryocytes, and germinal centre B cells [3]. The *BCL11A* gene is located on chromosome 2p16.1 and encodes an 835-amino acid protein. Q9H165-BC11A_HUMAN (UniProtKB/Swiss-Protein ID) is a Krüppel-like C2H2 zinc-finger protein. A total of 5,788 single-nucleotide polymorphisms (SNPs) in *BCL11A* are reported in the National Centre for Biotechnology Information (NCBI) database [4]. The SNPs often have observable characteristics or traits, and non-synonymous single nucleotide polymorphisms (nsSNPs) are mostly associated with inherited disorders in living organisms. These nsSNPs are located in the protein-coding regions and have deleterious or neutral effects on protein structure and function due to variation in the amino acid sequence, which affects the transcription factor binding affinity and mRNA transcription stability [5]. Wet lab studies of nsSNPs intended to identify their effects on the structure and function of proteins are difficult and very expensive. However, *in silico* analysis using various state-of-the-art tools is an effective method for studying nsSNPs, as most such tools are cost-effective and reliable [6–8]. The current study explored the list of reported nsSNPs in the *BCL11A* gene with the aim of predicting their deleterious effects on gene function, structure, and interactions with functional partners using various state-of-the-art *in silico* tools.

## Materials and methods

### Retrieval of SNPs data and protein sequence

The BCL11A protein sequence was downloaded from NCBI [(Accession ADL14508.1 UniProt database http://www.uniprot.org) (UniProtKB ID Q9H165-BC11A_HUMAN)] in May 2017. Minor allele frequencies and the list of reported SNPs were obtained from the NCBI dbSNP database (build 150) [4].

### Deleterious nature of coding nsSNPs

The functional impacts of nsSNPs were predicted using computational tools with *in silico* algorithms. SIFT (http://sift.jcvi.org/), PolyPhen-2 (http://genetics.bwh.harvard.edu/pp2), SNAP (https://www.rostlab.org/services/SNAP/), SNPs&GO (http://snps-and-go.biocomp.unibo.it/snps-and-go/), PANTHER (http://pantherdb.org/), PhD-SNP (http://snps.biofold.org/phdsnp/phd-snp.html), mCSM (http://bleoberis.bioc.cam.ac.uk/mcsm/), PROVEAN (http://provean.jcvi.org/index.php), and I-Mutant (http://folding.biofold.org/I-Mutant/I-Mutant2.0.html) were used to predict deleterious and neutral nsSNPs.

### SIFT for sequence homology

Sorts Intolerant from Tolerant (SIFT) is a sequence homology tool that predicts the negative effects of an amino acid substitution on protein function. The output gives the SIFT score; a SIFT score ≤0.05 corresponds to tolerated nsSNPs, and a SIFT score ≥0.05 indicates a deleterious nsSNPs [9].

### PolyPhen-2 for predicting amino acid substitutions

Polymorphism Phenotyping v2.0 (PolyPhen-2) is an online tool that predicts the possible effects of an amino acid substitution on the structure and function of a human protein. PolyPhen estimates the sensitivity and calculates the position-specific independent count (PSIC) score, which classifies coding nsSNPs as benign, possibly damaging, and probably damaging [10].

### mCSM for the stability of protein through atomic distance

The Mutation Cut-off Scanning Matrix (mCSM) server predicts atomic distance patterns surrounding an amino acid residue. Based on this, it predicts the impact of a nsSNP on the stability of the protein. The Protein Data Bank (PDB) format of BCL11A protein was provided as input, and the output score for each variant was determined to differentiate the destabilising variants [<0 (ΔΔG)] from the other variants [11].

### PROVEAN for predicting the impact on protein function

Protein Variation Effect Analyser (PROVEAN) is used to predict changes in the biological functions of the BCL11A protein due to an amino acid substitution [12]. A PROVEAN score less than ≤2.5 for a human protein variant is considered deleterious.

### SNAP2 to compare the solvent accessibility of the native and mutated proteins

SNAP2 (Screening for Non-Acceptable Polymorphisms 2) is a neural network-based classifier tool that predicts changes due to a nsSNP on the secondary structure, pathogenicity, and solvent accessibility of the protein. It differentiates among wild-type and non-synonymous SNPs based on their score (+100, strongly predicted to have an effect; –100, predicted to be neutral) [13].

### SNPs&GO for functional information and gene ontology

SNPs&GO is a web tool used to predict the impact of variation in BCL11A protein by computing the molecular function and functional information in the Gene Ontology (GO) database. Probability values >0.5 for each variant were predicted to indicate disease-causing nsSNPs [14,15].

### PANTHER tool for evolution-related protein function and stability

Protein Analysis through Evolutionary Relationships (PANTHER) is an online tool that compares the sequence of the BCL11A protein with those of a family of evolutionarily related protein sequences. It determines the probability of deleterious (*P*_*deleterious*_) nsSNPs based on the substitution position-specific evolutionary conservation (subPSEC) score [16]. If the subPSEC score is ≥0.5, the nsSNP is considered deleterious.

### Homology modelling and structural analysis

The three-dimensional (3D) structure of the BCL11A protein is not available in the PDB. Therefore, the 3D structure was constructed using a computational tool through a hierarchical approach with Iterative Threading Assembly Refinement (I-TASSER). I-TASSER selects the most significance (n = 10) threading templates [17] and works using the multiple threading approach in the program LOMETS. The FASTA sequence of BCL11A protein was submitted to the I-TASSER server to predict the confidence score (C-score), which was –0.76, and estimated the TM-score (0.62±0.14) and RMSD (10.3±4.6Å). The 3D model generated was subjected to structural validation using PROCHECK [18]. Mutant structures were generated using the Swiss-PDB Viewer and Pymol software (ver. 1, Schrodinger) [19]. Energy minimisation for the wild-type BCL11A and mutants was estimated using the program GROMACS [20].

### Computation of accessible surface area (ASA)

VADAR is a web-based tool (http://redpoll.pharmacy.ualberta.ca/vadar) used to quantify the quality of protein structures [21]. We submitted the BCL11A protein PDB file as input, and VADAR analysed the structural parameters for both individual residues and the entire protein. It outputs the accessible surface area (ASA) of a protein.

### Prediction of ligand binding site with COACH

COACH is a meta-server approach that combines multiple functional annotation results from the programs COFACTOR, TM-SITE, and S-SITE, based on the I-TASSER structure prediction. COACH was run using the protein FASTA sequence.

### SRide predicts the stabilising residues

The SRide online server was used to identify stabilising residues to differentiate mutant from wild-type BCL11A based on stabilising protein residues [22].

### Prediction of solvent accessibility of the protein

FlexPred is a web server that is used to determine the solvent accessibility of a BCL11A protein sequence to identify the residual positions involved in conformational switches and those potentially causing pathogenic disorders [23].

### HBAT for hydrogen bond analysis

The Hydrogen Bond Analysis tool (HBAT) was used to study the effects of hydrogen bond formation on the 3D structure of BCL11A protein and to explore variations between the wild-type and mutant BCL11A in terms of changes in the distances and angles between hydrogen bonds due to a change in an amino acid [24].

### Molecular dynamic simulation

DelPhi was used for molecular dynamic simulation to calculate the folding free energy and total difference in energy in the solvated condition of wild-type and mutated BCL11A proteins [25]. The PDB model structures of both the wild-type and mutant BCL11A were used as input to find the grid, solvation, and coulombic energies of the wild-type and mutated proteins.

### Protein–protein complex interactions

Protein–protein complex interactions were determined using STRING 10 (https://string-db.org/), Sride (http://sride.enzim.hu/), Prism (http://cosbi.ku.edu.tr/prism/index.php), and FlexPred (http://flexpred.rit.albany.edu/), as described elsewhere [5].

### Statistical analysis

The predictions of various state-of-the-art *in silico* computational tools were subjected to correlation analysis using SPSS v19. The significance of differences between the predictions of the various computational tools were compared using the Student’s *t*-test. A *p*-value <0.01 was considered significant.

## Results

### SNP dataset selection, screening and prediction of nsSNPs from the *BCL11A* gene

A total of 11463 SNPs were identified in the *BCL11A* gene (*Homo sapiens*) sequence through a dbSNP database search using the SNP option at variation Class. These included 266 non-synonymous, 189 coding synonymous, 6 nonsense, and 6 stop-gained SNPs, as well as 799 in 5′ the untranslated region (UTR) and 486 in the 3′ UTR. In the human population, 1% of the DNA sequences have variations that cause numerous disorders, such as sickle cell anaemia, diabetes, obesity, and thalassemia [26]. Polymorphisms in the exonic region have the ability to regulate the protein function, whereas SNPs in the promoter and intronic regions may change the transcription activity and splicing mechanism. Defects in the splicing region can change mRNA expression and affect protein function. Missense or nonsense variations cause changes in the protein-coding regions.

### Computational algorithms used to identify deleterious nsSNPs

**Fig 1:**
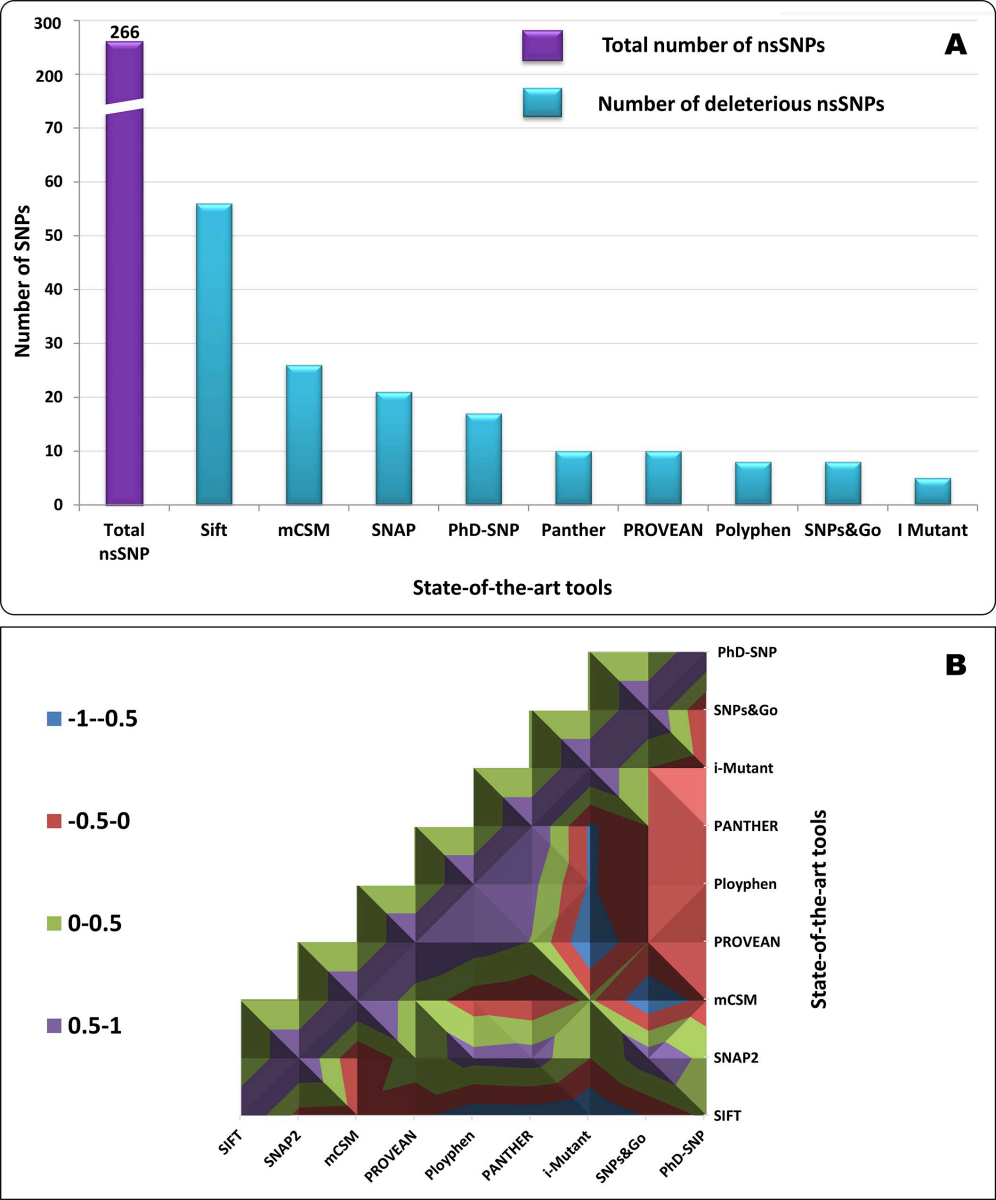
Screening for, and prediction of, deleterious nsSNPs in the *BCL11A* gene by different computational tools. A: Number of nsSNPs predicted as pathogenic by each state-of-the-art tool. B: Surface chart of the correlations among predictions of deleterious nsSNPs in the *BCL11A* gene by different computational tools.

**Fig 2:**
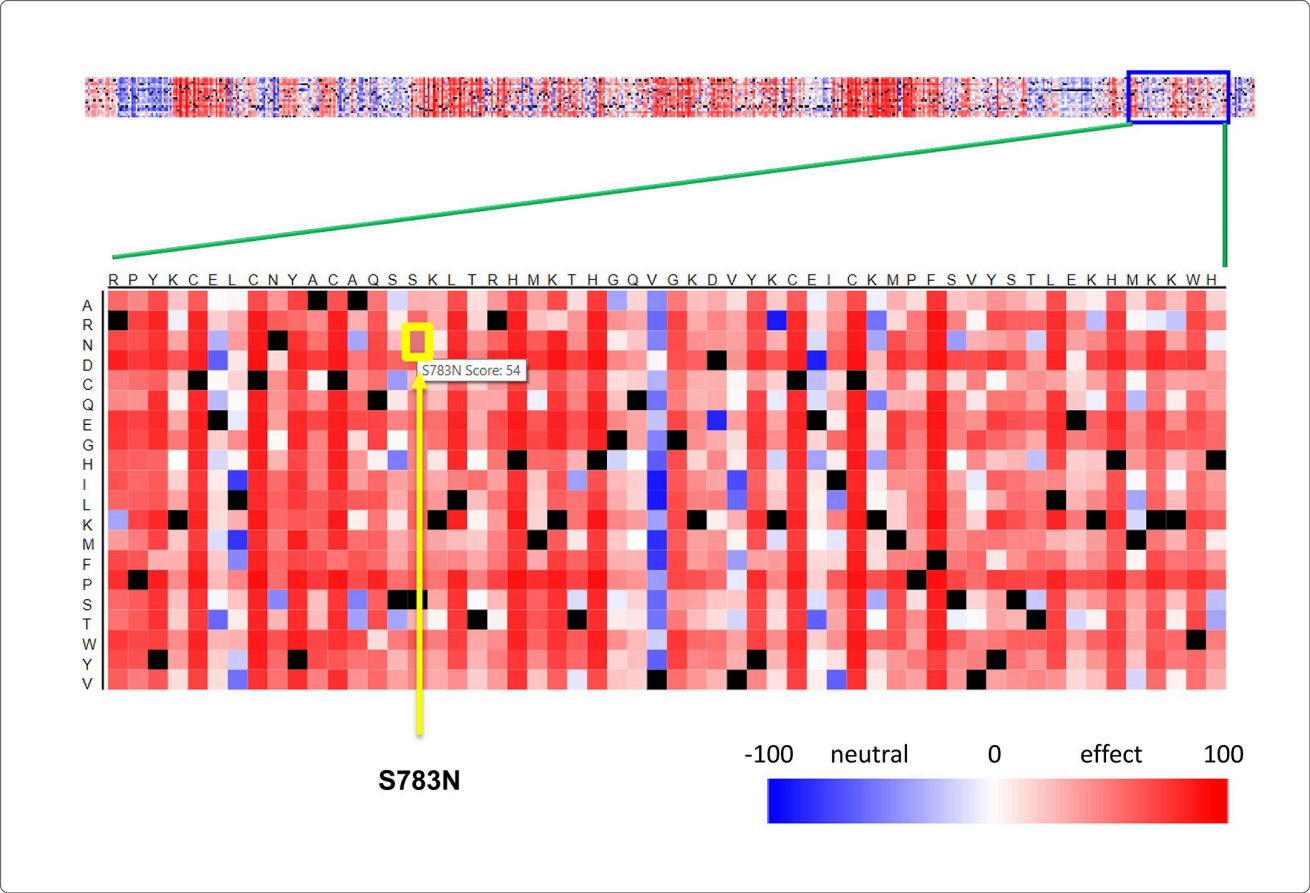
Heatmap of BCL11A protein. The yellow box indicates the most pathogenic amino acid change (S783N) in the *BCL11A* gene, corresponding to rs61742690.

To identify deleterious nsSNPs, we used nine *in silico* algorithms: SIFT, SNAP, PolyPhen-2, PANTHER, I-Mutant, PROVEAN, SNPs&GO, mCSM, and PhD-SNP (Fig 1). All nine algorithms were used separately to identify deleterious nsSNPs, and the results were then correlated. The percentage of deleterious nsSNPs varied with each tool. I-Mutant predicted the fewest nsSNPs in the *BCL11A* gene as damaging or deleterious non-synonymous polymorphisms, and SIFT predicted the most. The overall analysis and comparison of the predictions using these algorithms revealed that five nsSNPs were more deleterious. These five nsSNPs were the most frequently predicted as problematic by most of the computational tools. The nsSNP rs61742690 was predicted to be the most deleterious, as supported by eight of the nine state-of-the-art tools (Table 1). The BCL11A protein sequence in FASTA format was given as input data to SNAP2 using the GenBank sequence ADL14508.1 as the source data. SNAP2 predicted an amino acid change from serine to asparagine at the 783^rd^ position as a functionally effective change (Fig 2). The nsSNP rs17028351 was predicted to be deleterious by seven algorithms (77.78%), two nsSNPs (rs62142605 and rs74987258) were predicted as deleterious by six tools (66.67%), and the nsSNP rs115666026 was predicted as deleterious by five (55.56%) algorithms (Table 1). The correlations among the predictions of deleterious nsSNPs in the *BCL11A* gene by different computational tools were positive (Fig 1). The total number of nsSNPs found to be neutral or benign by each tool was as follows: 40 nsSNPs (66.6%) in SNAP, 43 nsSNPs (71.6%) in PhD-SNP, 50 nsSNPs (83.3%) in PANTHER, 52 nsSNPs (86.6%) in SNPs&GO, 52 nsSNPs (86.6%) in PolyPhen-2, 52 nsSNPs (86.6%) in I-Mutant, and 54 nsSNPs (90%) in PROVEAN.

**Table 1:** Details of genetic variation in the *BCL11A* gene predicted as high-risk SNPs out of 266 nsSNPs.

SNP ID	Amino acid variant	Single nucleotide variation	mCSM		PolyPhen		PANTHER		I-Mutant		SNPs&GO		SIFT		PROVEAN		SNAP2		PhD-SNP	
			score ΔΔG	Prediction	Score	Prediction	SubPSEC	Prediction	DDG<0	Prediction	Probability	Prediction	score	Prediction	Score	Prediction	Score	Prediction	Prediction	Reliability Index
**rs61742690**	S783N	C/T	–0.203	Destabilising	1	Probably damaging	750	Probably damaging	–0.94	Decrease Stability	0.638	Disease	0.002	Deleterious	–2.749	Deleterious	54	effect	Neutral	8
**rs62142605**	D643N	C/T	–0.112	Destabilising	0.999	Probably damaging	750	Probably damaging	–1.1	Decrease Stability	0.535	Disease	0.004	Deleterious					Neutral	4
**rs17028351**	G451S	C/T	–0.481	Destabilising	1	Probably damaging	750	Probably damaging	–0.62	Decrease Stability	0.52	Disease			–2.75	Deleterious			Disease	2
**rs115666026**	K670R	C/T	–0.179	Destabilising	0.897	Probably damaging	456	Probably damaging	–0.61	Decrease Stability		Neutral							Neutral	6
**rs74987258**	M313L	G/T	–2.025	Destabilising	0.992	Probably damaging	750	Probably damaging	–0.91	Decrease Stability		Neutral					18	effect	Neutral	6

### Homology modelling and structural analysis of the BCL11A protein

**Fig 3:**
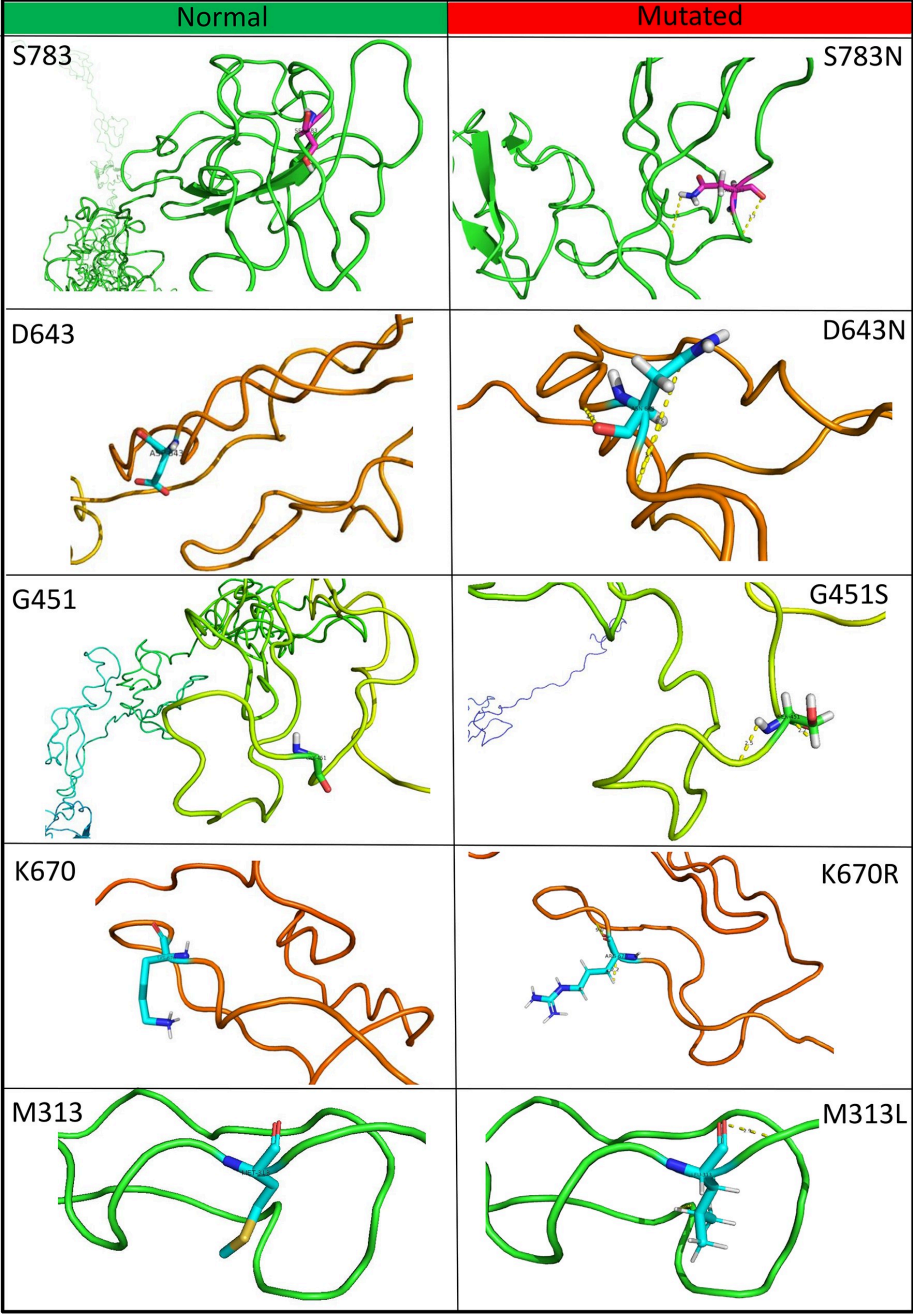
Structural models of the wild-type and mutated BCL11A proteins.

The I-TASSER server was used to predict the possible 3D structure of the wild-type BCL11A protein. The I-TASSER server generates various models based on the confidence of each model, which is quantified using the C-score. The best model was selected based on the best C-score (–0.76). Then, the BCL11A wild-type structure was mutated using the highly deleterious substitutions identified using the nine tools. To construct the mutant models of the rs17028351, rs62142605, rs74987258, rs115666026, and rs61742690 nsSNPs, Pymol was used (Schrodinger, LLC) (Fig 3). Each point mutation due to each SNP was generated in the native model of BCL11A protein separately, and then the mutated BCL11A model protein structure was refined using the SWISS-PDB viewer. Structural alterations due to mutations in BCL11A were confirmed using the 3D structure of the wild-type and mutated BCL11A using SWISS-PDB (S1 Fig). Energy minimisation was determined with GROMOS 96, which minimises the forces acting on each atom in a collection of atoms to obtain the most thermodynamically stable BCL11A structure. The final stable structure of BCL11A had an energy value of –19756 kJ/mol, compared with –10915 kJ/mol before the energy minimisation process (S2 Fig).

### Structural validation of the native and mutant models using a Ramachandran plot and PROCHECK analysis

**Fig 4:**
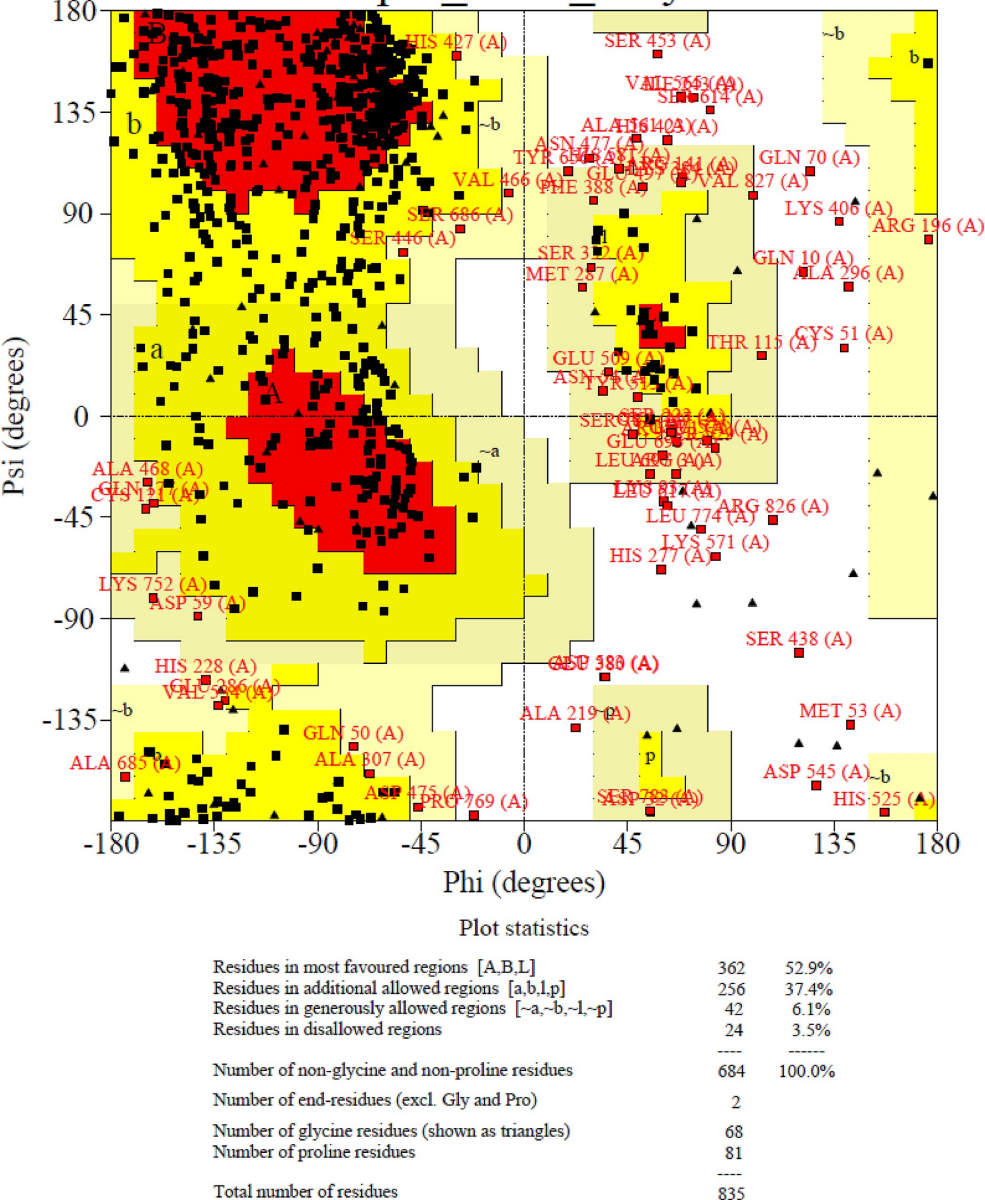
Ramachandran plot of BCL11A protein.

The structural validation and stereochemical properties of the predicted wild-type and mutated models of BCL11A protein were analysed using the SWISS-MODEL server [27]. A Ramachandran plot was drawn, and the structure was analysed by the PROCHECK algorithm in SWISS-MODEL. The Ramachandran plot revealed that the phi/psi angles of 52.9% of the residues fell in the most favoured regions, 37.4% of the residues were in additional allowed regions, 3.5% of the residues were in disallowed regions, and 6.1% fell in generously allowed regions (Fig 4). The constructed BCL11A protein had an estimated QMEAN6 score of 0.18 and Z-score of –6.45, clearly indicating that the 3D model was within acceptable standards. Further analysis of the secondary structure in the Ramachandran plot statistics indicated that the percentage of the most favoured regions was more than 50% in the predicted structure. The PROCHECK analysis indicated that the G factor value was >–0.76.

### Protein–protein interaction

**Fig 5:**
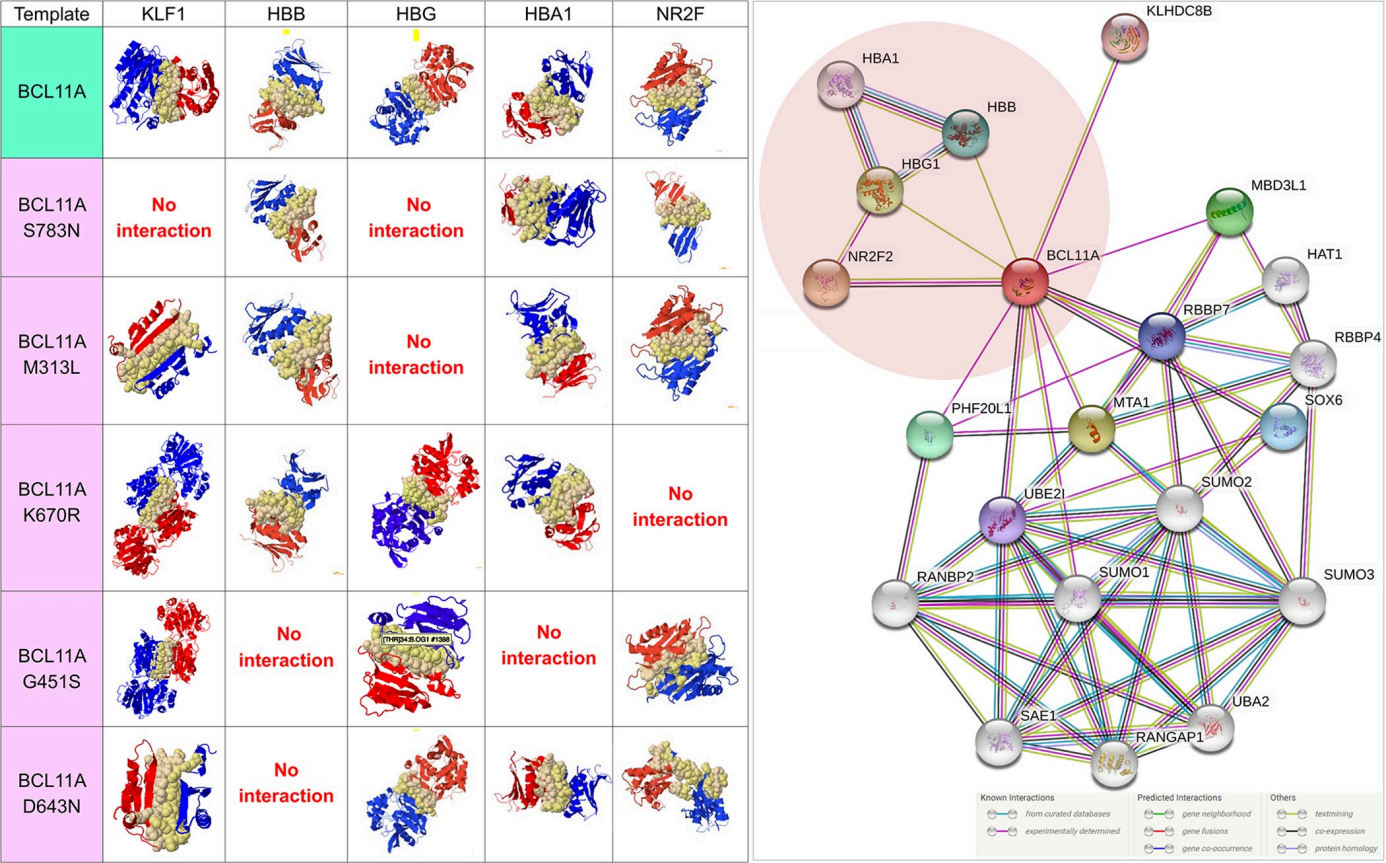
Interaction of the wild-type and mutated BCL11A proteins. Left: protein–protein interaction. The green row indicates the interactions between wild types. The pink rows indicate the interaction between the mutated BCL11A and wild-type proteins (HBB, HBG, HBA1, or NR2F). Right: Functional association network of the BCL11A protein. The region in brown indicates the proteins of interest associated with the foetal haemoglobin level.

**Fig 6:**
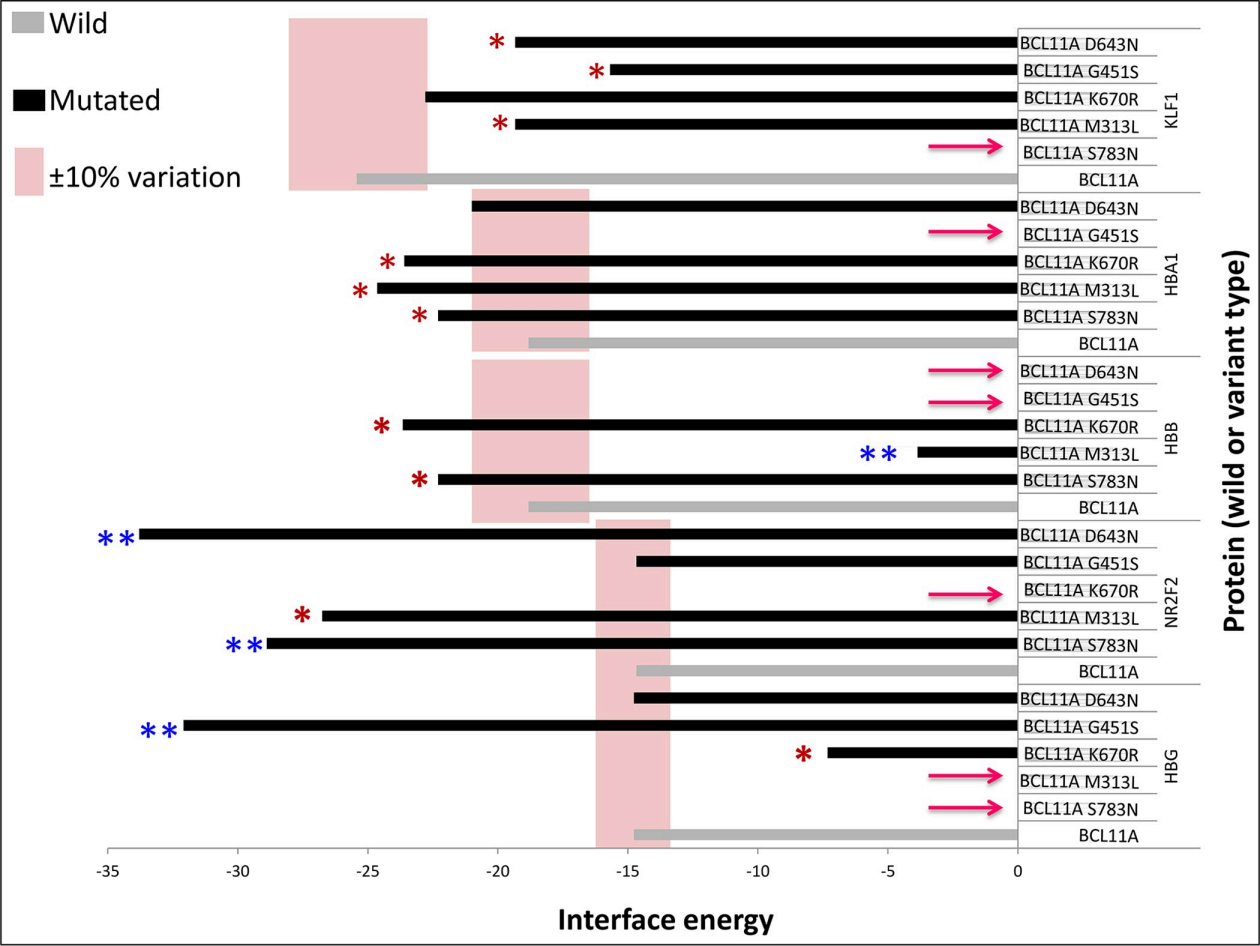
Interface energy of the template–target protein complex. An arrow indicates no interaction between the mutated BCL11A and wild-type proteins (KLF1, HBB, HBG, HBA1, or NR2F). The vertical shaded areas indicate ±10% variation in the interface energy of template–target protein complex compare with the wild-type. * Significant: indicates ±10% variation in the interface energy of the template–target protein complex compared with the wild-type protein interaction. ** Highly significant: indicates ±100% variation in the interface energy of the template–target protein complex compared with the wild-type protein interaction.

**Fig 7:**
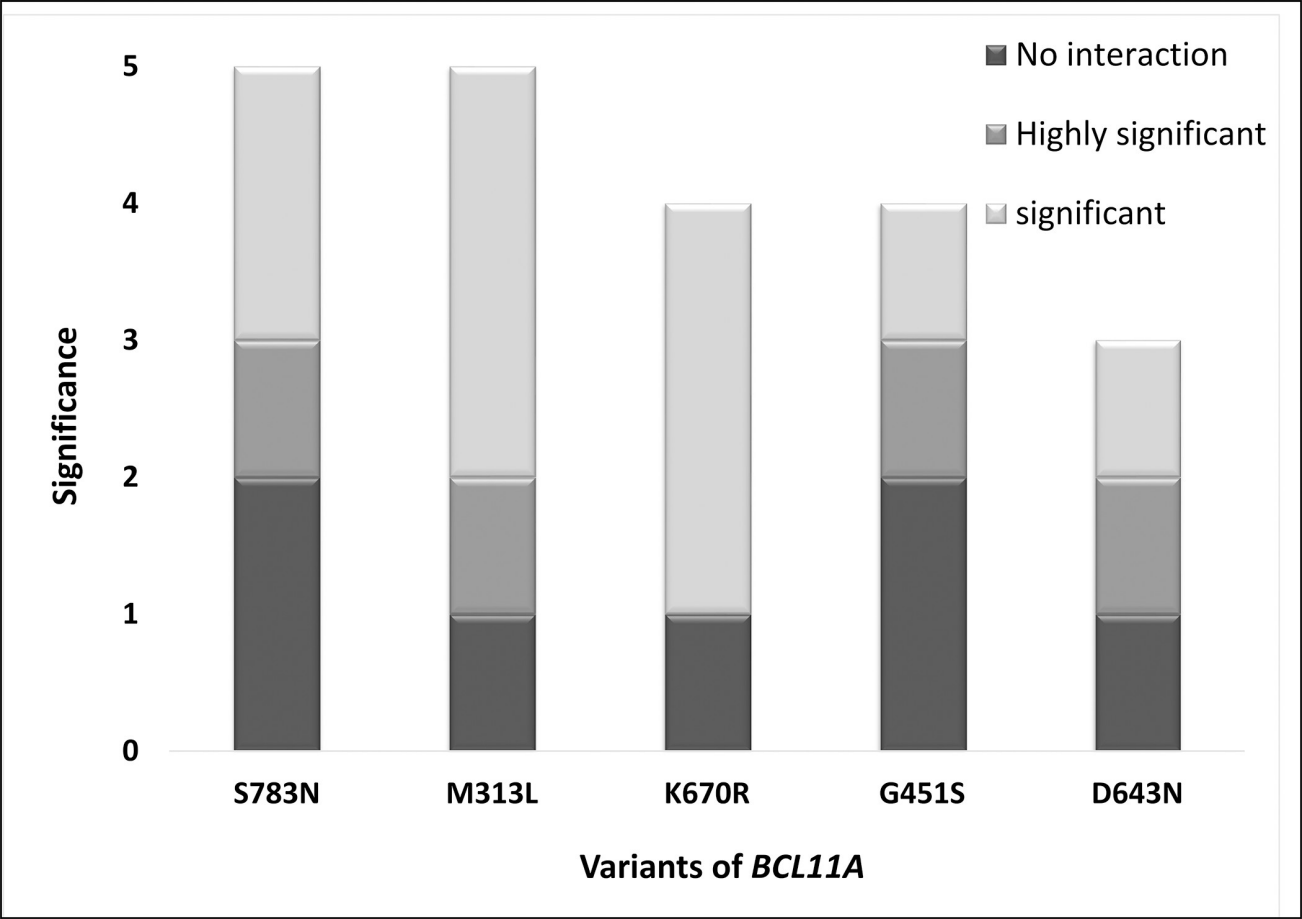
Overall significance of the variation in the protein–protein interaction and changes in the interface energy of the template–target protein complex.

**Fig 8:**
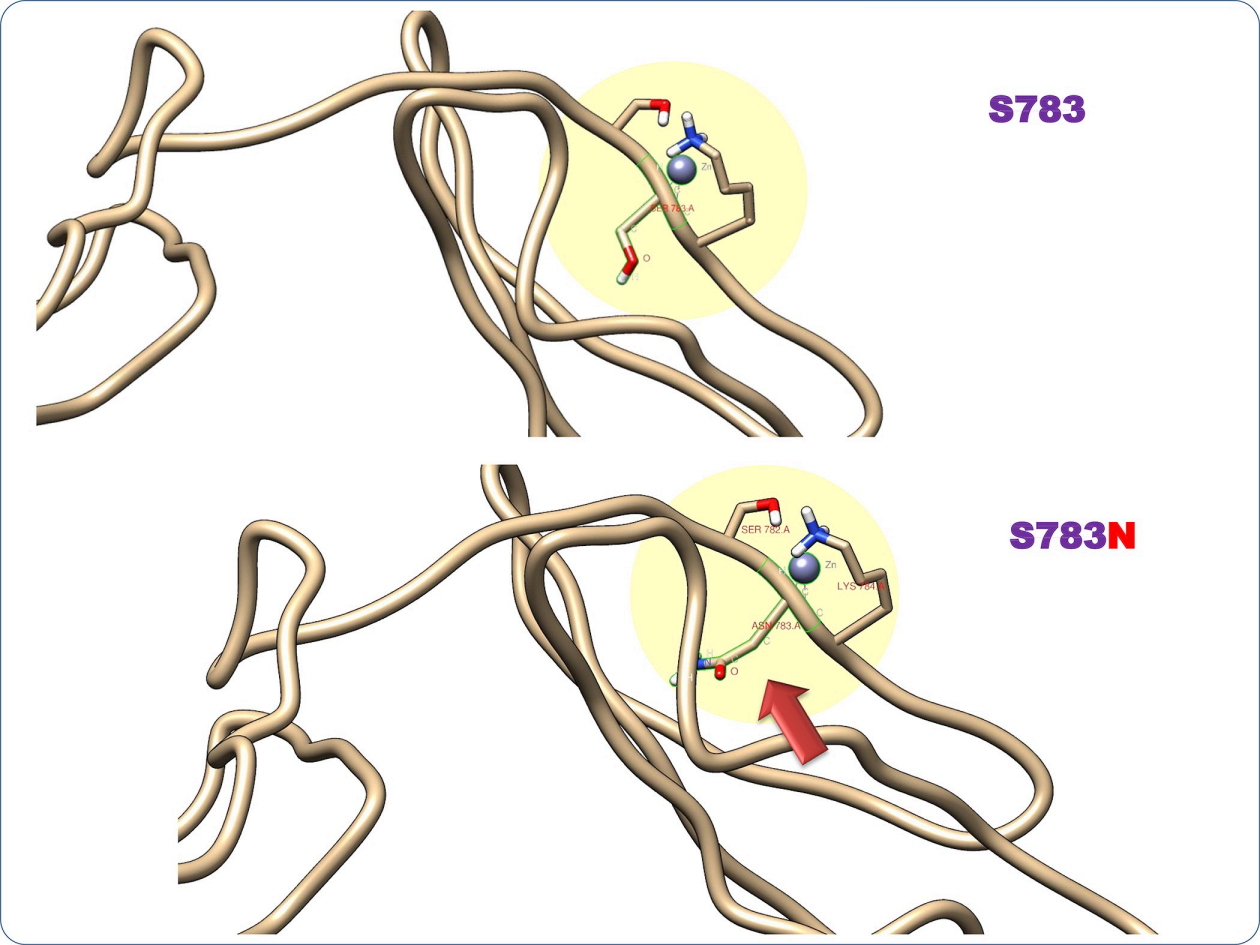
Structural change in the zinc-finger domain of BCL11A due to nsSNP rs61742690 (S783N).

The interactions between the functional partners (KLF1, HBB, HBG, HBA1, or NR2F) of BCL11A involved in haemoglobin synthesis and the foetal-to-adult switch based on STRING and the literature (Fig 5) were analysed. The results showed complete failure of the interaction between protein molecules due to the amino acid change (Fig 5). The interface energy of the template (wild-type or mutated BCL11A) and target protein (KLF1, HBB, HBG, HBA1, or NR2F) complex was calculated (Fig 6). The deviation (±10% or ±100%) in the interface energy due to the amino acid substitution in the BCL11A protein while interacting with the target protein (KLF1, HBB, HBG, HBA1, or NR2F) was calculated. Highly significant (±100% variation) deviation in the interface energy due to the amino acid substitution was observed for four interactions (Fig 6). Furthermore, the cumulative significance of the various protein–protein interactions between the functional partners (KLF1, HBB, HBG, HBA1, or NR2F) and template (wild-type or mutated BCL11A) indicates that the S783N amino acid substitution was the most significant of the five most pathogenic nsSNPs (S783N, D643N, G451S, K670R, and M313L; Fig 7; S1 Table). The most deleterious (nsSNP rs61742690) is in the zinc finger domain of the BCL11A protein, which involves amino acids 770 to 792. Hence, the impact of nsSNP rs61742690 on the zinc-finger domain of the BCL11A protein was analysed. A change in the structure of the zinc-finger domains due to nsSNP rs61742690 (S783N) was seen (Fig 8).

## Discussion

An elevated level of foetal haemoglobin (HbF, α2γ2) ameliorates the severity of sickle cell disease (SCD) and β-thalassemia (β-thal). The HbF level is highest at birth, and it is replaced by the adult haemoglobin (α2β2) within 1 year. The reactivation of the production of HbF is one strategy for the treatment of SCD and β-thal. Researchers have been concentrating on the permanent reactivation of foetal globin expression by mediating the machinery that silences foetal globin production by interfering with the regions associated with the hereditary persistence of HbF, such as BCL11A, HBS1L-MYB, and the β-globin locus itself. These regions were identified through a genome-wide association study [2]. The present study examined the effects of SNPs on the structural and interactive behaviours of BCL11A protein using state-of-the-art *in silico* tools. The sequential application of these tools has been used to screen the most pathogenic polymorphisms in various genes [5,28]. The present study also used the sequential application of these tools to identify deleterious variants in *BCL11A*; these might interfere with the machinery silencing HbF production. Altogether, we screened 11,463 SNPs in the *BCL11A* gene in various dbSNP databases for their impact on its structure and interaction with various protein molecules. The pathogenicity of 266 retrieved non-synonymous SNPs for screening tests was considered using various *in silico* tools. This approach identified five nsSNPs as highly pathogenic: rs61742690, rs62142605, rs17028351, rs115666026, and rs74987258. This is a smaller number than predicted previously in various genes using the same tools [5,28]. This finding eliminates the enormous amount of laboratory work required to screen pathogenic nsSNPs. Further, the molecular dynamic simulation and homology modelling studies of the mutated proteins (S783N, D643N, G451S, K670R, and M313L) revealed their functional and structural impacts [29]. The most pathogenic nsSNP, based on the protein–protein complexes interaction, was rs61742690. These analysis showed clear evidence in the 3D model of the BCL11A protein indicating that the 3D model of BCL11A protein is acceptable [18].

Protein–protein complex interaction studies help to elucidate the mechanisms of protein signalling, protein binding, regulation of biological functions, and effects of substitution mutations on function [29–32]. Protein–protein complex interaction studies reduce the need for complex experimental studies and can be used on a large scale [5,28,29,33–36]. The calculated interface energy of the BCL11A protein was –547.92, which explains the non-bonded atomic interactions in the protein model. The reliability score of the model was calculated using the QMEAN6 score. The quality of the model was estimated and was found to be compatible with better protein models with higher values [37]. The constructed BCL11A protein was longer than the *BCL11A-L* isoform; hence, it is clear that the constructed BCL11A protein is the *BCL11A-XL* isoform (extra-long isoform of BCL11A) [38]. The more efficient and less error-prone *in silico* methodologies revealed that rs61742690, which involved an S783N amino acid change in the zinc-finger domain of BCL11A, is an appropriate target for disrupting the BCL11A-mediated foetal-to-adult globin switch to continue the production of the HbF in SCD and β-thalassemia patients. The *in-silico* data obtained from bioinformatics analyses should be validated in future experiments that reflect its biological context.

## Conclusion

nsSNP rs61742690, which corresponds to an S783N amino acid change, was predicted to be the most deleterious among 266 non-synonymous SNPs in *BCL11A*, as confirmed by eight of the nine computational tools employed. The complete failure in the protein–protein complex interaction and significant changes in the interface energy with functional partners such as KLF1, HBB, HBG, HBA1, or NR2F revealed that rs61742690 (S783N) in the zinc-finger domain is a suitable target for disrupting BCL11A-mediated foetal-to-adult globin switching.

## Supporting information

S1 Fig Superimposed structures of the BCL11A mutant models. (PNG)

S2 Fig Homology modelling structure of BCL11A; red indicates the zinc finger. (PNG)

S1 Table Predicted hot spots for interface residues of the template–target protein complex. (DOCX)
