# Supplementary figures and images for: The rs61742690 (S783N) single nucleotide polymorphism is a suitable target for disrupting BCL11A-mediated foetal-to-adult globin switching
Source: PLoS One. 2019 Feb 15;14(2):e0212492. doi: 10.1371/journal.pone.0212492 (PMC6377191; doi:10.1371/journal.pone.0212492)

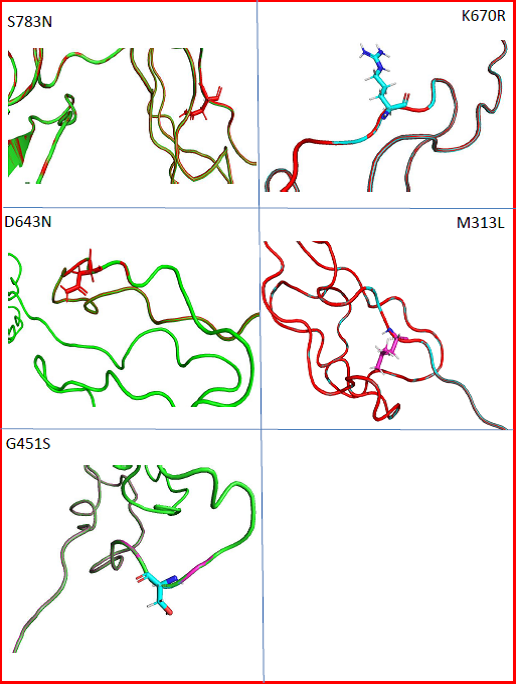

Supplement: S1 Fig — (PNG) [file pone.0212492.s001.png]

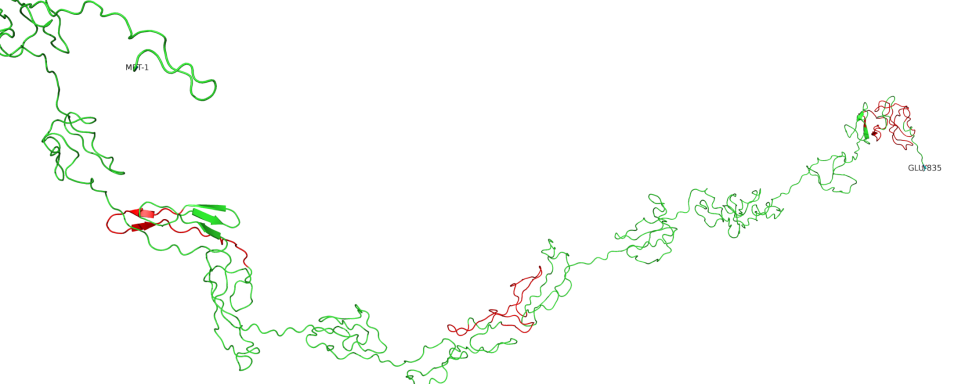

Supplement: S2 Fig — (PNG) [file pone.0212492.s002.png]
