# Supplementary material for: The rs61742690 (S783N) single nucleotide polymorphism is a suitable target for disrupting BCL11A-mediated foetal-to-adult globin switching
Source: PLoS One. 2019 Feb 15;14(2):e0212492. doi: 10.1371/journal.pone.0212492 (PMC6377191; doi:10.1371/journal.pone.0212492)
Supplement: S1 Table — (DOCX) [file pone.0212492.s003.docx]

**S1 Table 1. Predicted hot spots interface residues of the template-target protein complex.**

| **Template** | **Target** | **Hot spot** |
| --- | --- | --- |
| **BCL11A** | HBG | 247LEU, 250ILE, 254GLU, 255LEU, 256HIS, 257ILE, 258PHE, 2247LEU, 2250ILE, 2254GLU, 2255LEU, 2256HIS, 2257ILE, 2258PHE |
| **BCL11A 783** |  | No interaction |
| **BCL11A 313** |  | No interaction |
| **BCL11A 451** |  | 24ILE, 28SER, 30ILE, 31ALA, 32ARG, 35ALA, 38TYR, 41TYR, 49GLU, 50PRO, 51LEU, 52PRO, 70TYR, 24ILE, 28SER, 30ILE, 31ALA, 32ARG, 35ALA, 38TYR, 41TYR, 42GLN, 49GLU, 50PRO, 51LEU, 52PRO, 70TYR |
| **BCL11A 643** |  | 247LEU, 250ILE, 254GLU, 255LEU, 256HIS, 257ILE, 258PHE, 2247LEU, 2250ILE, 2254GLU, 2255LEU, 2256HIS, 2257ILE, 2258PHE |
| **BCL11A 670** |  | 247LEU, 250ILE, 254GLU, 255LEU, 256HIS, 257ILE, 258PHE, 2247LEU, 2250ILE, 2254GLU, 2255LEU, 2256HIS, 2257ILE, 2258PHE |
| **BCL11A** | NR2F2 | 14VAL, 15LEU, 18LEU |
| **BCL11A 313** |  | 14VAL, 15LEU, 18LEU |
| **BCL11A 670** |  | No interaction |
| **BCL11A 451** |  | 14VAL, 15LEU, 18LEU |
| **BCL11A 643** |  | 22GLU, 23LEU, 29LEU, 33ALA, |
| **BCL11A 783** |  | 25SER, 41LEU, 43HIS, 60LEU, 100ASP |
| **BCL11A** | HBB | 276LEU, 278GLN, 286ALA, 289LEU, 292GLN, 293LEU, 294LEU, 266ASP, 268ASP, 282GLY, 285ARG, 286ALA, 289LEU, 290VAL, 293LEU, 294LEU |
| **BCL11A 313** |  | 276LEU, 278GLN, 286ALA, 289LEU, 292GLN, 293LEU, 294LEU, 266ASP, 268ASP, 282GLY, 285ARG, 286ALA, 289LEU, 290VAL, 293LEU, 294LEU |
| **BCL11A 451** |  | No interaction |
| **BCL11A 670** |  | 276LEU, 278GLN, 286ALA, 289LEU, 292GLN, 293LEU, 294LEU, 266ASP, 268ASP, 282GLY, 285ARG, 286ALA, 289LEU, 290VAL, 293LEU, 294LEU |
| **BCL11A 643** |  | No interaction |
| **BCL11A 783** |  | 276LEU, 278GLN, 286ALA, 289LEU, 292GLN, 293LEU, 294LEU, 266ASP, 268ASP, 282GLY, 285ARG, 286ALA, 289LEU, 290VAL, 293LEU, 294LEU. |
| **BCL11A** | HBA1 | 276LEU, 278GLN, 286ALA, 289LEU, 292GLN, 293LEU, 294LEU, 266ASP, 268ASP, 282GLY, 285ARG, 286ALA, 289LEU, 290VAL, 293LEU, 294LEU. |
| **BCL11A 313** |  | 276LEU, 278GLN, 286ALA, 289LEU, 292GLN, 293LEU, 294LEU, 266ASP, 268ASP, 282GLY, 285ARG, 286ALA, 289LEU, 290VAL, 293LEU, 294LEU. |
| **BCL11A 670** |  | 276LEU, 278GLN, 286ALA, 289LEU, 292GLN, 293LEU, 294LEU, 266ASP, 268ASP, 282GLY, 285ARG, 286ALA, 289LEU, 290VAL, 293LEU, 294LEU. |
| **BCL11A 643** |  | 276LEU, 278GLN, 286ALA, 289LEU, 292GLN, 293LEU, 294LEU, 266ASP, 268ASP, 282GLY, 285ARG, 286ALA, 289LEU, 290VAL, 293LEU, 294LEU. |
| **BCL11A 451** |  | No interaction |
| **BCL11A 780** |  | 276LEU, 278GLN, 286ALA, 289LEU, 292GLN, 293LEU, 294LEU, 266ASP, 268ASP, 282GLY, 285ARG, 286ALA, 289LEU, 290VAL, 293LEU, 294LEU. |
| **BCL11a** | KLF1 | 183ALA, 237GLN, 262ILE, 263THR, 236, LEU, 237GLN, 271ALA, 275ALA |
| **BCL11A 783** |  | No interaction |
| **BCL11a 313** |  | 80ILE, 81ASP, 82GLU, 64HIS, 68LEU, 74VAL. |
| **BCL11a 670** |  | 271ILE, 272ILE, 276LEU, 278CYS, 283ALA, 286PHE, 287ILE, 289SER, 290ILE, 293GLU, 299ARG, 271ILE, 272ILE, 276LEU, 278CYS, 279GLU, 283ALA, 286PHE, 287ILE, 289SER, 290,ILE, 293GLU, 299ARG, 309PHE. |
| **BCL11a 451** |  | 271ILE, 272ILE, 276LEU, 278CYS, 283ALA, 286PHE, 287ILE, 289SER, 290ILE, 293GLU, 299ARG, 271ILE, 272ILE, 276LEU, 278CYS, 279GLU, 283ALA, 286PHE, 287ILE, 289SER, 290 ILE, 293GLU, 299ARG, 309PHE. |
| **BCL11a 643** |  | 80ILE, 81ASP, 82GLU, 64HIS, 68LEU, 74VAL. |
